# The Effect of Child Trauma on the Relation between Psychological Well-Being and Depressive Symptoms in Chilean University Students

**DOI:** 10.3390/healthcare10122463

**Published:** 2022-12-06

**Authors:** Paulina Barros, Rodrigo Assar, Alberto Botto, Caroline Leighton, Yamil Quevedo, Juan Pablo Jiménez

**Affiliations:** 1 Departamento de Psiquiatría y Salud Mental Oriente, Facultad de Medicina, Universidad de Chile, Santiago 7500922, Chile; 2 Instituto de Ciencias Biomédicas, Facultad de Medicina, Universidad de Chile, Santiago 8380453, Chile; 3 MIDAP Millennium Institute for Research in Depression and Personality, Santiago 7820436, Chile

**Keywords:** childhood trauma, psychological well-being scale, depression, mental health

## Abstract

(1) Background: There is consistent evidence of the impact of early adverse experiences on mental health in adulthood, especially as a risk factor for depression. However, their influence on positive aspects of mental health such as well-being has been less extensively studied. Therefore, this study aims to investigate the effect of traumatic childhood experiences on the relationship between depression and psychological well-being in a sample of university students. (2) Methods: The Childhood Trauma Questionnaire—Short Form (CTQ-SF), the Beck Depression Inventory (BDI-IA), and Ryff’s psychological well-being scale were administered to 700 Chilean university students. Several regression models were used to analyze the interaction between variables, with multivariate SEM being applied to hierarchize the relationships found. (3) Results: Emotional Neglect and Abuse stand out as the types of maltreatment with the greatest impact on mental health, associated first with a decrease in the self-acceptance dimension of psychological well-being and then with depressive symptomatology in adulthood. (4) Conclusions: Results provide evidence that early trauma has an important impact on mental health, increasing the risk of depression, however, its impact is greater on positive aspects of health, such as self-acceptance, a fundamental element in the construction of psychological well-being.

## 1. Introduction

The World Health Organization (WHO) proposes understanding mental health as a state of complete physical, mental, and social well-being and not simply as the absence of mental illness. This definition recognizes that mental health consists of positive elements, highlighting well-being as a central element in understanding health. Thus, the presence or absence of a mental disorder does not determine a person’s level of mental health, which explains why some people with mental illness may report high levels of well-being; likewise, merely having no mental disorders does not guarantee optimal mental health. From this perspective, positive mental health and mental illness can be interpreted as distinct phenomena that ’run on different tracks’ but may cross paths [1].

Exposure to early adverse events in childhood is one of the phenomena with the greatest impact on mental health. The complexity of this concept has made it difficult to generate a widely accepted definition; however, the proposal of the seminal CDC-Kaiser Permanente Study [2] has been the most widely used in multiple lines of research. This study defined Adverse Childhood Experiences (ACEs) as all experiences of abuse (physical, emotional, and sexual) and neglect (physical and emotional), such as family dysfunction, growing up with a family member with a mental illness, parental divorce, or domestic violence, to name but a few.

There is widespread evidence that these early adverse events, experienced recurrently over long periods, affect several dimensions of child development, including neurobiological, cognitive, affective, and general health ([3,4,5]). The high prevalence of child maltreatment makes it a global problem; according to WHO data, 25% of the adult population reports having experienced physical abuse [6]. Exposure to childhood trauma has long-term consequences, such as the appearance of various diseases in adulthood and, recently, in old age. For example, it has been linked to inflammatory, autoimmune, and chronic diseases ([7,8]). In addition, it has been strongly linked to increased risk for anxiety, mood, behavioral, and substance disorders ([7,9,10]), as well as to suicidal ideation [11].

Studies widely highlight the role of childhood trauma as a major risk factor for developing and maintaining depression in adulthood ([9,12]). In addition, depressive episodes tend to occur more frequently and are more persistent, affecting the course of the disease and hindering treatment success ([9,13,14,15]). Interestingly, particularly in young adulthood (18 to 23 years old), an increase in these trauma-associated depressive symptoms has been observed; its effect is considered direct at this age before later stages of adulthood in which various stressors may interfere ([16]). Furthermore, the literature describes the mediating role of cognitive emotion regulation strategies in this relationship, particularly in university students ([17,18]).

Regarding the specific effects associated with different forms of maltreatment, evidence highlights that those involving threats, such as physical and emotional abuse, are more strongly related to poor mental health outcomes ([16,19]). Furthermore, studies conclude that psychological abuse and neglect are the types of maltreatment most strongly associated with depressive outcomes ([9,11,19]). For example, individuals who reported emotional abuse in childhood have been found to exhibit a nearly three-fold increase in their risk of depressive disorder over their lifespan, with the risk being higher for female than male subjects [12].

The consequences of emotional abuse and neglect in childhood have also been shown to affect positive aspects of health, such as general well-being ([20,21]). The concept of well-being can be understood from two main perspectives: hedonic and eudaimonic. The former is related to the concept of subjective well-being, which refers to the pursuit of pleasure and a sense of satisfaction with life, while the concept of psychological well-being (eudaimonic well-being) is related to optimal human functioning [22]. Several studies indicate that early adverse experiences cumulatively contribute to diminished well-being in adulthood ([11,23]). However, due to the conceptually diverse notions of well-being used in this research, it is difficult to identify the specific consequences of early trauma in this regard.

Within the eudaimonic tradition, Carol Ryff proposes a multidimensional model that allows us to operationalize psychological well-being into six distinct components: self-acceptance, personal growth, purpose in life, positive relationships with others, environmental mastery, and autonomy. Some factors related to the study of psychological well-being, such as self-regulation, life experiences, and personality traits, may be affected as a consequence of early adverse experiences [22]. Research on this topic is scarce.

In Chile, as well as in other occidental countries, university life stands out as a period of exploration and transition to build personal identity [24]. During this period, there are several challenges, such as achieving greater autonomy, searching for a life project, and meeting one’s and one family’s expectations, to name a few. All these factors can contribute positively to the achievement of psychological well-being and, at the same time, strongly challenge the adaptive resources of students, becoming a very significant stressor event that would explain the higher incidence of depressive conditions in this population [25].

According to recent literature reviews, there is sufficient evidence confirming a negative inverse relationship between psychological well-being and depression in several age groups: adults, older adults, and adolescents ([26,27]). Moreover, well-being is also considered an outcome of depression treatments, with people who exhibit higher baseline psychological well-being responding better to depression treatment [28]. In light of these results, well-being and psychological distress seem to be associated with a unidimensional concept of mental health, where both operate as extremes of the same continuum. However, according to the two-continua model [29], mental health is a more complex balance than the description and categorization of pleasurable experiences or the absence of negative affect. More evidence is needed to understand the multidimensional mental health approach regarding early trauma.

Thus, this study aims to identify and measure the impact of early childhood traumatic experiences on the mental health of university students through their effect on the negative and positive aspects of mental health, such as depressive symptomatology and psychological well-being. We hypothesized that early trauma affects both dimensions of mental health, with a greater impact on its negative aspects, such as depressive symptoms. In addition, we expect to shed light on how these three variables (early trauma, psychological well-being, and depressive symptoms) can shape mental health.

## 2. Materials and Methods

### 2.1. Participants

In the present study, we employed a non-probabilistic sample of 700 male and female undergraduates at two Chilean universities, whose ages ranged from 18 to 30 years old. One university is in the capital city, and the other is in the south. The sample was not randomly designed, but no additional criteria were considered.

### 2.2. Procedure

We used a non-experimental, cross-sectional, exploratory, and descriptive design. As described in [27], university students were contacted by researchers who explained the objectives of the study and invited them to participate voluntarily. The universities were chosen for convenience. The researchers of our team are part of either the university of the capital or the one of the south. Those who accepted signed an informed consent approved by the ethics committees of both universities with procedures following ethical standards based on the Declaration of Helsinki.

The participants completed a self-administered battery of instruments using an Internet platform. Data were automatically organized in a private database. In this article, we focus on:

1. Ryff’s psychological well-being scale: Likert self-report scale composed of 29 items representing 6 dimensions. Spanish adaptation of D. van Dierendonck [22,30].
2. Beck’s depression inventory (BDI-IA): Self-report instrument designed to assess depressive symptomatology in adults and adolescents. It consists of 21 items, evaluated on a 4-point Likert scale [31].
3. Childhood Trauma Questionnaire-Short Form (CTQ-SF): A 28-item self-report inventory that measures the severity of multiple types of childhood trauma trough the subscales of Emotional Abuse, Physical Abuse, Sexual Abuse, Emotional Neglect, and Physical Neglect. It does not register the time the trauma occurred [32].

In addition, we considered demographic data (sex and age).

### 2.3. Data Analysis

We statistically analyzed results as follows:

- (BDI,Ryff) outliers. Given the (BDI,Ryff) linear model, these are the individuals whose |standardizedresidual|>2 [33].
- Hypothesis testing. ANOVA and Student’s *t*-test for means comparison were used to compare scores in different conditions. Pearson’s correlation tests was used to measure the linear association between two-dimensional or final scores.
- Linear regression models. Simple, additive, and interaction models were fitted, computing $R^{2}$ as the measure of linear fitting and, for significance of coefficient estimates, *t*-test for comparing two means.
- Structural equation modeling (SEM). Multilevel models were built with the aim of fitting hierarchical relationships between variables. Model fit is analyzed using comparative fit index (CFI, acceptable over 0.9), toot mean square error of approximation (RMSEA, acceptable less than 0.06), and standardized root mean square residuals (SRMR, acceptable less than 0.08).

We considered the Ryff score as the dependent variable and the BDI, CTQ dimensions, sex, and age as independent, explanatory variables. For the SEM approach, there are two levels. The BDI score may depend on the other mentioned independent variables, and Ryff depends on the BDI and the remaining variables.

All computations and analyses were performed using RStudio V1.2 (R V3.6.3). In particular, for structural equation modeling (SEM), we used the R Package lavaan [34].

## 3. Results

**Figure 1:**
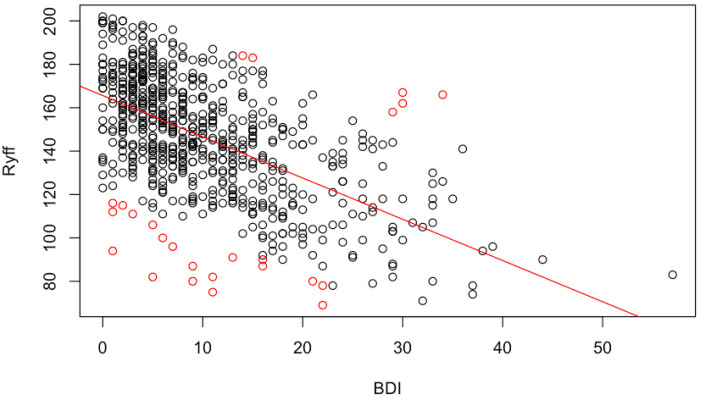
Ryff score as a function of BDI score. The straight red line marks the linear tendency and red circles the (BDI,Ryff) scores classed as outliers. Red points below the straight line belong to individuals whose Ryff score is too low for the BDI that they exhibit, while those over the straight line represent individuals with excessively high Ryff levels.

From 700 participants, we eliminated 1 subject with scarce data and 25 (BDI,Ryff) outliers (Figure 1). Most of them are subjects with Ryff scores that are too low relative to their BDI scores. Thus, we restricted the analysis to these 674 participants.

Regarding demography, 55.9% are men and 44.1% women, aged between 18 and 30 years old at the time of sampling (mean = 20.5 and standard deviation = 1.6). Age does not depend on sex or university. The scores for the CTQ dimensions of Emotional Abuse, Sexual Abuse, Emotional Neglect, and Physical Neglect differ by sex. Emotional Abuse and Sexual Abuse are significantly higher in women than in men. Emotional Neglect and Physical Neglect are higher in men. The scores for the Ryff dimensions of Positive Relations and Life Purpose are the most dependent on sex. Both are significantly higher in women than in men. For the institutions, the capital city university (58.2%) and south university (41.8%) differ significantly in sex distribution (52% are women in the capital and 61.3% in the south university). The scores for the CTQ dimensions of Physical Neglect, Sexual Abuse, and Physical Abuse are higher in the university from the southern city than those from the capital; this fact is significant in general and for men. The scores for the Ryff dimensions of Life Purpose and Personal Growth are significantly the lowest in women from the southern city. More information is in Supplementary Table S1.

### 3.1. CTQ, BDI, and Ryff

General statistics of each instrument, including their dimensions, are shown in Table 1. We include information about cases in each range: Mild or Low, Moderate, and Severe or High.

Emotional Abuse is significantly more prevalent than other abuse dimensions (*p*-value zero for software). Sexual Abuse is the least common type, ranking lower than Physical Abuse (*p*-value <0.001). Regarding neglect, this emotional type is also the most common (*p*-value zero for software); see Table 1. Cut-off values for CTQ ranges depended on dimensions and were obtained from [35,36]. Emotional Abuse is the dimension with the largest number of cases in the severe range (5.1%), with scores of 16 or higher; furthermore, it exhibits the most cases in the moderate or severe range (12.9%). The most strongly correlated dimensions are Physical and Emotional Neglect (0.58), while Emotional Neglect and Sexual Abuse exhibit a weaker association (0.11). It is also worth noting that CTQ Emotional Abuse is correlated with CTQ Physical Abuse (0.52); see Table 2.

The Ryff scores range from 71 to 202, with an average of 147.4 and a standard deviation of 26.07, being slightly higher for women (*p*-value 0.27). Regarding the dimensions of the Ryff scale, Self-acceptance ranges from 4 to 28, with a mean of 21.07; Positive relations from 7 to 35, with a mean of 25.81; Purpose in life between 6 and 35, with a mean of 26.24; Autonomy from 7 to 42, with a mean of 26.72; Environmental mastery from 10 to 35, with a mean of 24.57; and Personal growth from 8 to 28 with a mean of 23.05. Because there are no clear thresholds for Ryff’s scale, we define the ranges in terms of number of SDs under the mean; Mild: [(Mean−SD),Mean), Moderate: [(Mean−2∗SD),(Mean−SD)), and Severe: <(Mean−2∗SD). Positive relations and Environmental mastery exhibit the highest percentage of moderate or severe cases (18.7%), while Self-acceptance has more severe cases (4.7%). The most strongly correlated dimensions are Self-acceptance and Purpose in life (Pearson’s correlation 0.80), followed by Environmental mastery and Self-acceptance (0.74). The least strongly correlated dimensions are Autonomy and Personal growth (0.29), followed by Autonomy and Positive relationships (0.32).

The BDI ranges from 0 to 57, with an average of 10.2 and a median of 8. BDI scores differ significantly by sex, being higher for women (*p*-value 0.0007). The BDI is considered high for values of 14 or more [31], and we do not consider intermediate ranges. Of all the participants, 23.9% (185 cases) have a high BDI score, with this percentage increasing to 31.3% for women.

### 3.2. BDI as a Function of CTQ

**Figure 2:**
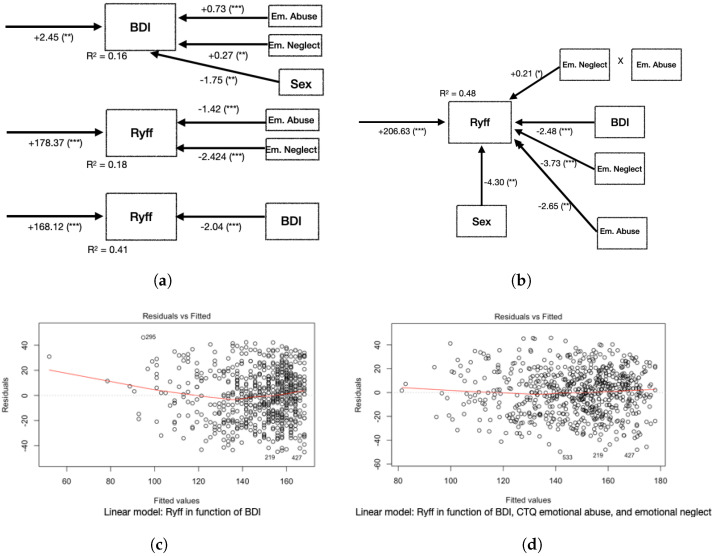
Models and residuals for the BDI and Ryff scores. (**a**) Diagrams of linear regression results for the BDI as a function of CTQ emotional dimensions and sex, Ryff as a function of CTQ emotional dimensions, and Ryff as a function of the BDI. (**b**) Linear regression for Ryff as a function of CTQ emotional dimensions, BDI, and sex. In the rows, we show coeff.(signif.) values, with notation: 0 *** 0.001 ** 0.01 * 0.05. (**a**) Diagram: Ryff as a function of the BDI. (**b**) Diagram: Ryff as a function of the BDI and emtional dimensions of CTQ. (**c**) Residuals: Ryff as a function of the BDI. (**d**) Residuals: Ryff as a function of the BDI and emtional dimensions of CTQ.

CTQ dimensions show positive correlations with BDI scores. From most to least correlated, they are Emotional Abuse (0.38), Emotional Neglect (0.25), Physical Neglect (0.15), Physical Abuse (0.15), and Sexual Abuse (0.13); see Table 2. All of them are significant. See Figure 2a for linear regression results.

### 3.3. Ryff as a Function of CTQ

We analyzed the associations between CTQ dimensions and Ryff dimensions, focusing on the most significant associations and checking the effect of non-concordance. The CTQ Emotional Neglect is the most strongly correlated with Ryff’s Self-Acceptance (−0.39), CTQ Emotional Abuse with Ryff’s Environmental mastery (−0.38), CTQ Physical Neglect with Ryff’s Environmental mastery (−0.26), CTQ Physical Abuse with Ryff’s Environment mastery (−0.14), and CTQ Sexual Abuse correlates more with Ryff’s Environmental mastery (−0.14); see Table 2.

### 3.4. Ryff as a function of BDI and CTQ

For correlations between all the pairs of dimensions and final scores, see Table 2. As expected, the highest correlations were observed for dimensions of the same instrument, which we analyzed instrument by instrument. The strongest associations between different instruments were found for Ryff and the BDI (−0.64 for overall scores; −0.62 for Self-acceptance with BDI scores).

In a hierarchical sense, Ryff–BDI associations were the strongest, followed by associations between CTQ and Ryff, which were stronger overall than those between CTQ and the BDI. This fact strengthens our idea of explaining the psychological status first in terms of the Ryff scale, considering its association with the BDI, as the manifestation of depressive symptomatology, and with CTQ dimensions, as evidence of early traumatic experiences, which could explain well-being and psychological distress beyond the presence or absence of disease.

A (BDI,Ryff) linear model shows, with $R^{2}=0.41$ and linear correlation −0.64, that when the BDI increases by one unit, the Ryff decreases by 2.04 units; see Figure 1 and Figure 2a.

**Figure 3:**
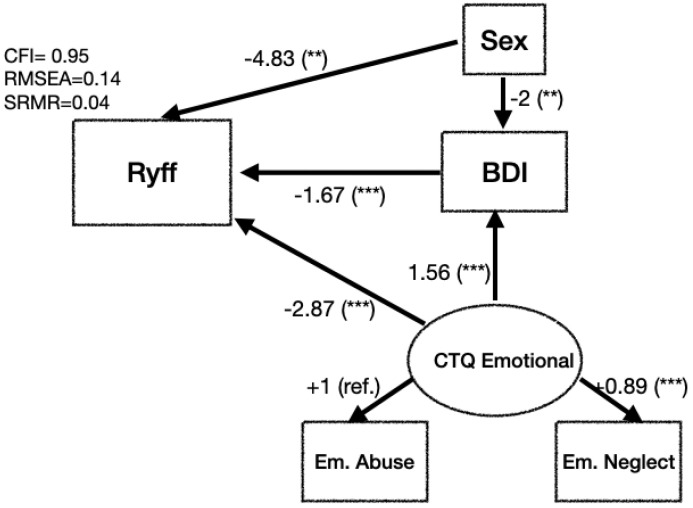
Results of structural equation modeling for Ryff and BDI. Emotional dimensions of CTQ define a latent variable that we labeled CTQ Emotional. We show results for linear models using coeff.(signif.) values, with notation: 0 *** 0.001 ** 0.01.

Regarding the inclusion of CTQ in the model (BDI,Ryff), we considered the Ryff score as a function of the BDI score but added the most relevant dimensions of the CTQ as explanatory variables; that is, the Ryff model as a function of the BDI and its interactions with the CTQ Emotional Abuse and CTQ Emotional Neglect and sex. This model achieves an $R^{2}$ of 0.48, with more significant effects for the BDI (coefficient −2.48), CTQ Emotional Neglect (coefficient −3.73), and CTQ Emotional Abuse (coefficient −2.65). As an alternative approach, we explored structural equation modeling (Figure 3). This revealed that the emotional dimensions of the CTQ impact significantly on the BDI (coefficient −1.56) and Ryff (−2.97) scores. The BDI has a highly significant impact (coefficient −1.67, *p*-value <0.001) on Ryff, while sex impacts less significantly (*p*-value <0.01) on the BDI and Ryff.

## 4. Discussion

First, let us discuss some remarkable results.

According to the linear model that we obtained for (BDI,Ryff), each one-unit increase in the BDI entails a 2.48-unit decrease in the Ryff, each one-unit increase in CTQ Emotional Neglect leads to a 3.73-unit decrease in the Ryff, and each one-unit increase in CTQ Emotional Abuse results in a 2.65-unit decrease in the Ryff. The interaction (product) between Emotional Abuse and Emotional Neglect is significant, too (coefficient 0.21); see Figure 2b Sex is also significant: if the participant is a man, the Ryff decreases by 4.3 units. A comparison of residuals shows that the inclusion of the CTQ’s emotional dimensions leads to a decrease in residuals and improves fit, in particular for extreme Ryff values (Figure 2c,d).

Due to strong associations with Emotional Abuse and Emotional Neglect, as well as with the BDI (Table 2), we also produced a linear model for Ryff Environmental mastery as a function of the BDI. It strongly improved by including the interaction with CTQ Emotional Abuse and with CTQ Emotional Neglect. As a result, $R^{2}$ increases from 0.38 to 0.44, with the most significant effects being for the BDI (coefficient −0.46, *p*-value <0.001), CTQ Emotional Neglect (coefficient −0.72, *p*-value <0.001), and CTQ Emotional Abuse (coefficient −0.61, *p*-value <0.01). As for the general model, the interaction between Emotional Abuse and Emotional Neglect is significant, too (coefficient 0.041, *p*-value <0.05). Sex is not a significant factor. Given these findings, continued exploration of this topic is warranted. As additional information, we included findings from simple linear regression on the influence of each sociodemographic characteristic and childhood trauma dimension on psychological well-being and depressive symptoms (Supplementary Table S2).

Our structural equation modeling results allow us to measure the differential impact of early adverse emotional episodes on depression symptoms and well-being (Figure 3). The model indicated that the CTQ’s emotional dimensions have a significant impact on both well-being and depression symptoms. Even though RMSEA=0.14, the model has acceptable model fit (CFI=0.95, SRMR=0.04). Including other dimensions of childhood trauma causes the model to greatly lose fit.

Let us discuss the strengths and limitations of our study.

We consider that a strength of this study is the methodological and statistical support of our approaches. The consideration of a wide range of methodologies allowed us to obtain results with evidence in the data. In particular, the SEM approach has been seldomly used in this context, and it allowed us to consider the BDI and Ryff at different levels and, with that, we can approach them separately. Another important strength to highlight is the large sample used and its sociodemographic characteristics, particularly because it included students from two universities located in different regions of the country, central and southern, which could have represented very different realities due to the urban–rural characteristics that distinguish them.

As a limitation, it is impossible to attribute early trauma effects to the population studied. Linear regression and SEM are procedures used for hypothesis modeling and not for causal modeling. Therefore, more complete models can be found, and the inclusion of other variables cannot be excluded. Moreover, as the study has a cross-sectional design, it should be confirmed later by longitudinal or comparative studies with another age range. Because the sample was not randomly designed, although no additional criteria were considered, representativeness is not assured.

Regarding how our results compare with other research findings, it is worth noting that the emotional dimension of childhood has been found to have the strongest impact on BDI scores [37]. The literature also shows that personality aspects (mostly neuroticism) mediate the effect of childhood abuse on depression symptoms. These findings can help us to complete the structural equation modeling presented in this article. In this context, it would be interesting to explore the effects of personality on psychological well-being and whether this indirect relationship also appears in well-being modeling. Social relationships are also relevant to explore. Furthermore, ref. [38] identifies other mediators of the effects of childhood abuse on depression, the most salient of which are resilience and school connectedness.

Now, let us discuss the scope of our results.

At the qualitative level, our analysis confirmed our hypothesis of an additional association with childhood trauma; in particular, that emotional experiences impact not only depression symptoms but also well-being. In fact, Emotional Neglect shows stronger associations with Ryff than with the BDI (−0.38 versus 0.25). As shown in Figure 2a, the model for Ryff as a function of Emotional Abuse and Emotional Neglect fits better than the model for the BDI as a function of the same variables; also, the inclusion of the CTQ’s emotional dimensions impacts positively on the (BDI,Ryff) model, shown in Figure 2a. The latter model allows us to measure the differential impact of depression symptoms and early adverse episodes on well-being.

First, it was observed that early trauma is associated with greater depressive symptomatology; in particular, the Emotional Abuse dimension, followed by Emotional Neglect, proved to have the greatest impact on this association [9]. In line with these findings, ref. [12] found that these two dimensions of trauma show the strongest association with depression. In a previous study, ref. [39] found that people who reported Emotional Abuse in childhood had an almost threefold increase in the risk of presenting a depressive disorder in the life cycle, with this risk being greater in women than in men. Consistently, a positive and significant correlation between Emotional Abuse and Emotional Neglect was found in association with neuroticism [40], a personality trait widely studied due to its strong link to depression ([41,42,43]).

Second, the present study also showed the effect of early trauma on well-being levels. This effect is independent of the effect on depressive symptomatology and is comparatively greater. In line with previous findings [44], Emotional Neglect was the dimension of early trauma found to have the greatest negative effect on well-being. However, our results further identify that Emotional Neglect impacts well-being to a greater extent through its negative effect on Self-acceptance. In this regard, it can be presumed that not responding to children’s fundamental emotional needs forces them to handle situations that require skills belonging to more advanced developmental stages. It is very likely that, due to immaturity, they fail to resolve these adverse situations or resolve them at a high emotional cost, which leads to the development of a diminished and questioned self-image; this damage to the self can become a secondary stressor that affects development and threatens well-being in adulthood [45]. Reasons why this effect of early adverse experiences is said to be direct in early adulthood: research with university students has shown that this population has a special cognitive vulnerability; they would have a deficit in their cognitive emotional regulation strategies, which can moderate the impact of early trauma on a current depression [18].

When comparing the specific effect of Emotional Abuse and Neglect on depression and well-being, it is observed that the model of psychological well-being as a function of Emotional Abuse and Emotional Neglect fits better than the model of depressive symptomatology as a function of the same variables. This result suggests that both dimensions of trauma, taken together, have greater negative consequences on psychological well-being. With respect to these results, it can be hypothesized that Emotional Neglect leads to the development of a more abandoning self that impedes the achievement of well-being, and that Emotional Abuse produces a more punishing self that permanently attacks self-esteem with hyper-criticism, which could also affect well-being and ultimately promote depression.

According to the stress generation hypothesis [46], individuals are not passive recipients of events in the world around them; on the contrary, they are active agents in shaping their environment and their daily experiences. In this sense, certain personality traits, as well as behavioral and cognitive patterns, can lead the subject to be exposed to stressful situations that can cause the onset of depressive symptoms, partly explaining the recurrence of depression. In relation to child abuse, it has been reported that Emotional Abuse, but not Sexual or Physical Abuse, prospectively predicts greater stress generation, which is mediated by negative inferential styles [47]. These findings suggest that targeting negative cognitive styles in clinical settings, especially in patients with a history of childhood Emotional Abuse, may be important in reducing the occurrence of negative life events, which may decrease the risk of depression recurrence.

Taking all this into account, it is interesting to observe that early trauma affects positive mental health (well-being) more than mental illness. This result can be explained by the two-continua model, where the presence/absence of mental illness and mental health are different but related phenomena [29]. Our results indicate that the dimensions of early trauma strengthen the inverse relationship between depressive symptomatology and psychological well-being, mainly affecting depressive symptomatology. Thus, an increase in depressive symptomatology results in slightly more than a two-fold decrease in psychological well-being levels. At the same time, Emotional Neglect decreases psychological well-being almost four times as much, while Emotional Abuse affects it twice as much. This negative effect on well-being increases even more if the participant is male. These results allow us to conclude that people who have experienced Emotional Abuse and/or Neglect tend to exhibit lower levels of well-being and greater depressive symptomatology. Thus, Neglect and Emotional Abuse in childhood were found to be the dimensions of early trauma with the greatest impact on mental health in adulthood.

These results were reinforced by our structural equation modeling, which allowed us to separately measure the differential impact of early emotional episodes on depression symptoms and well-being. The model shows, with an acceptable fit, that the emotional dimensions of early trauma significantly affect both aspects of depression and psychological well-being. Thus, early emotional events indirectly affect well-being through the manifestation of depressive symptoms, but this is not the only way: early emotional events affect it directly. Our findings account for the significant impact that early trauma has on mental health, not only because it increases the risk of suffering depressive symptomatology but also because it is associated with lower levels of well-being. Thus, early trauma, and in particular the dimensions of Emotional Abuse and Neglect, negatively influence two key aspects of mental health: positive functioning and illness.

In our view, the observation that Emotional Abuse and Neglect have an impact on well-being that is independent of depressive symptomatology is also consistent with the two-continua model of mental health, which proposes two orthogonal dimensions: absence or presence of mental health and absence or presence of mental illness [29]. A recent study that explored this model using a sample of African college students found three latent classes: languishing with moderate endorsement of depressive symptoms, flourishing with low endorsement of depressive symptoms, and moderate mental health with high endorsement of depressive symptoms [48]. Early adversity may not only be considered a risk factor for mental illnesses but may also have a direct impact on well-being. In this regard, a study that employed a sample of Eritrean college students found that the presence of multiple early adversities and domestic violence can predict both psychological distress and subjective well-being, with these effects being mediated by current stressful events [49].

With respect to mental health intervention strategies, the results of this study highlight the importance of promoting parental skills and bonding in childhood with the aim of reducing Emotional Abuse and Neglect. At a therapeutic level, our results suggest that, when suffering is deemed to be a product of neglect or Emotional Abuse, therapy goals may benefit from working on self-acceptance and the development of a sense of control over the external world. A limitation of our study is that the instrument used to measure depression (BDI-IA) only evaluates depressive symptoms, without offering depression diagnoses. Therefore, subjects with scores greater than 14 may not necessarily have clinical depression. Moreover, the other instruments used to assess child trauma and psychological well-being are self-report scales, which, as previously discussed [50], may be affected by recall bias or temperamental factors, which prior experiences can influence. In other words, these instruments may not evaluate childhood trauma but rather the “perception of childhood maltreatment”, thus failing to pass the gene–environment correlation (rGE) test. In other words, a personality trait or prior experience determines how the person perceives the environment rather than interacting with the environment to produce a new result.

## 5. Conclusions

This study provided evidence of the effect of early trauma on positive and negative aspects linked to mental health. Contrary to expectations, a greater impact of early trauma on psychological well-being was observed, particularly on the dimension of Self-acceptance. Emotional Neglect and Abuse stand out as the types of maltreatment with the greatest impact on health, decreasing levels of well-being and increasing depressive symptoms.

It is important to highlight the contribution of this study to the mental health of young university students. Considering the high rates of depression in this population and the evidence of the impact that the different stressors of university life have on them, it is very important to plan efficient and effective interventions to prevent and promote mental health in this group. According to the results of this study, developing tools that contribute to Self-acceptance can be a very efficient strategy for improving mental health.

In theoretical terms, this study contributed evidence to the two-continua model, showing that mental health’s positive and negative aspects interact differently, although in a related way, with vulnerability factors. The remaining challenge is to discover where, if at all, the two continua meet.

## Figures and Tables

**Table 1:** Clinical characterization of study participants (N=674). Annotated cases (percentage), Mean, SD, and cases (percentage) per severity range. For the Ryff scale, ranges are obtained in terms of number of SD over the mean.

Feature		N (%)	Mean (SD)	Mild Range (%)	Moderate Range (%)	Severe Range (%)
CTQ						
	Emotional Abuse	664 (98.5%)	8.47 (3.59)	9–12 (22.9%)	13–15 (7.8%)	≥16 (5.1%)
	Physical Abuse	664 (98.5%)	6.32 (2.28)	8–9 (9.8%)	10–12 (4.5%)	≥13 (3.2%)
	Sexual Abuse	664 (98.5%)	5.90 (2.25)	6–7 (12.7%)	8–12 (9.5%)	≥13 (2.9%)
	Emotional Neglect	664 (98.5%)	8.44 (3.35)	10–14 (24.5%)	15–17 (5.4%)	≥18 (1.2%)
	Physical Neglect	664 (98.5%)	6.33 (2.04)	8–9 (6.2%)	10–12 (7.5%)	≥13 (1.4%)
BDI						
	Score	674 (100%)	10.18 (8.24)	-	-	≥14 (23.9%)
	Men score	297 (44.1%)	9.05 (8.04)	-	-	≥14 (22.6%)
	Women score	377 (55.9%)	11.07 (8.29)	-	-	≥14 (31.3%)
Ryff						
	Score	663 (98.4%)	147.4 (26.07)	31.2%	14.5%	3%
	Men score	292 (43.3%)	146.7 (25.3)	32.5%	14%	3.1%
	Women score	369 (54.7%)	148 (26.69)	30.6%	14.9%	3%
	Self-acceptance	663 (98.2%)	21.07 (5.16)	33%	9.4%	4.7%
	Positive Relations	663 (98.4%)	25.81 (6.27)	26.4%	15.5%	3.2%
	Autonomy	663 (98.4%)	26.72 (7.15)	33.9%	12.2%	2.9%
	Env. Mastery	663 (98.4%)	24.57 (5.37)	29.3%	17.3%	1.4%
	Life Purpose	663 (98.4%)	26.24 (6.23)	29.1%	13.3%	4.2%
	Personal Growth	663 (98.4%)	23.05 (4.06)	35.4%	10.3%	3.2%

**Table 2:** Correlations between CTQ, BDI, and Ryff dimensions.

	(1)	(2)	(3)	(4)	(5)	(6)	(7)	(8)	(9)	(10)	(11)	(12)	(13)
(1) e. abuse	1	0.52	0.29	0.45	0.36	−0.31	−0.27	−0.14	−0.38	−0.27	−0.12	−0.33	0.38
(2) p. abuse	0.52	1	0.21	0.27	0.3	−0.08	−0.14	−0.01	−0.14	−0.1	−0.06	−0.12	0.15
(3) s. abuse	0.29	0.21	1	0.11	0.2	−0.10	−0.13	−0.09	−0.14	−0.09	−0.05	−0.13	0.13
(4) e. neglect	0.45	0.27	0.11	1	0.58	−0.39	−0.29	−0.12	−0.36	−0.36	−0.25	−0.38	0.25
(5) p. neglect	0.36	0.3	0.20	0.58	1	−0.2	−0.23	−0.08	−0.26	−0.23	−0.18	−0.25	0.15
(6) s. accept.	−0.31	−0.08	−0.1	−0.39	−0.2	1	0.51	0.43	0.74	0.8	0.59	0.87	−0.62
(7) relations	−0.27	−0.14	−0.13	−0.29	−0.23	0.51	1	0.32	0.53	0.43	0.41	0.71	−0.43
(8) autonomy	−0.14	−0.01	−0.09	−0.12	−0.08	0.43	0.32	1	0.45	0.33	0.29	0.65	−0.4
(9) Env. mastery.	−0.38	−0.14	−0.14	−0.36	−0.26	0.74	0.53	0.45	1	0.71	0.51	0.85	−0.61
(10) purpose	−0.27	−0.10	−0.09	−0.36	−0.23	0.80	0.43	0.33	0.71	1	0.54	0.82	−0.5
(11) grow	−0.12	−0.06	−0.05	−0.25	−0.18	0.59	0.41	0.29	0.51	0.54	1	0.69	−0.4
(12) Ryff	−0.33	−0.12	−0.13	−0.38	−0.25	0.87	0.71	0.65	0.85	0.82	0.69	1	−0.64
(13) BDI	0.38	0.15	0.13	0.25	0.15	−0.62	−0.43	−0.4	−0.61	−0.5	−0.4	−0.64	1

## Data Availability

Data supporting reported results will be made available during review process.

## Supplementary Materials

The following supporting information can be downloaded at: https://www.mdpi.com/article/10.3390/healthcare10122463/s1. Table S1: Sociodemographic characteristics and effects on psychological well-being, depressive symptoms, and childhood trauma; Table S2: Findings from simple linear regression on the influence of each sociodemographic characteristic and childhood trauma dimension on psychological well-being and depressive symptoms.
